# A Newborn with Panhypopituitarism and Seizures

**DOI:** 10.1155/2017/4364216

**Published:** 2017-02-01

**Authors:** Trupti Kale, Rachit Patil, Ramesh Pandit

**Affiliations:** ^1^Department of Pediatrics, Saint Anthony Hospital, Chicago, IL, USA; ^2^Department of Neurology, Medical College of Wisconsin, Milwaukee, WI, USA

## Abstract

Interstitial deletions on the short arm of chromosome 20 are uncommon, and therefore the clinical phenotype is poorly defined. Very few cases have been reported in the literature so far. In this report, we describe a 4-month-old female with a heterozygous deletion at 20p11.21p12.1 with panhypopituitarism and cardiac, gastrointestinal, and genitourinary anomalies along with dysmorphic facial features. We compared and discussed similar cases with overlapping deletions in 20p11 region. We wish to report this rare occurrence as this may better define the phenotypes of the 20p interstitial deletion with certain dysmorphic features, multiorgan involvement, and related clinical characteristics in this patient population.

## 1. Introduction

In this report, we discuss an interesting case of an infant who presented with a seizure after birth and was later found to have panhypopituitarism with an absence of pituitary gland, situs inversus, and left ureterocele with hydronephrosis. Chromosomal microarray analysis revealed a deletion at 20p11.21p12.1, spanning approximately 8.348 Mb. Deletion of 20p associated with panhypopituitarism is very uncommon, so we wish to report this rare occurrence. The aim of this case report is to further the understanding of the phenotypic features associated with interstitial deletions of chromosome 20 and offer a basis for the development of a possible syndromic profile for these deletions in the future.

## 2. Case Presentation

A female infant was born to a 28-year-old mother who had scant prenatal care. She was born by normal vaginal delivery at 39 weeks of gestation, with Apgar scores of 9 at 1 min and 9 at 5 min. Her birth weight was 3046 grams. On physical exam, she was noted to have excessive hair on the forehead, flat bridge of nose, retrognathia, and bitemporal flattening. She had 2/6 systolic murmur along the left sternal border. She did not need any resuscitation initially, but, at about 24 hours of life, she developed tonic clonic convulsions and was found to have hypoglycemia of 26 mg/dL. EEG completed at that time revealed diffuse slowing, which was thought to be due to effect of phenobarbital, which had been given after first seizure.

Soon after, the baby developed abdominal distention and bilious vomiting. An upper GI study with small bowel follow through showed situs inversus, malrotation, annular pancreas, and duodenal obstruction. The stomach was displaced towards the right and the duodenum crossed the midline, with the jejunal loops located in the left upper quadrant of the abdomen. The patient underwent exploratory laparotomy with Ladd's procedure and duodenoduodenostomy was performed.

Initially, the hypoglycemia was managed with additional dextrose containing intravenous fluids. In spite of a glucose infusion rate of 11.3 mg/kg/min, the patient's hypoglycemia persisted. Serum levels of insulin and growth hormone were measured, with next episode of hypoglycemia. Serum insulin was 0.91, which ruled out hyperinsulinemia. Growth hormone (GH) was 0.519, suggesting GH deficiency. ACTH stimulation test was normal. MRI of the brain including dynamic sellar protocol revealed congenital absence of the pituitary gland, with neurohypophysis and adenohypophysis. An ECHO revealed biventricular hypertrophy, mild peripheral pulmonary stenosis, and small patent ductus arteriosus with left to right shunting. A bone survey did not show the hemivertebrae or other bony abnormalities. The abdomen and pelvic ultrasounds were done, which showed situs inversus and polysplenia. The body of the stomach and the spleen were found in the right upper quadrant.

A renal ultrasound revealed bilateral enlargement of kidneys, left hydronephrosis with a duplicated collecting system, and obstruction of the upper pole moiety by ureterocele, causing moderate hydroureteronephrosis. A vesicocystourethrogram was negative for reflux. Cystoscopy revealed a left ureterocele filling over half of the bladder. A Lasix renal scan was done, which revealed the left kidney contributing 47.89% and the right kidney contributing 52.11% of the total renal function, suggestive of a partial functional obstruction of left kidney. Chromosomal microarray analysis was done and results were as described later in report.

After being diagnosed with panhypopituitarism, this patient was started on prednisone, levothyroxine, and growth hormone medications. She was started on antibiotic prophylaxis to prevent urinary tract infection. Later, ureterocele was incised due to large degree of hydronephrosis, hydroureter, and risk of infection. After discharge from neonatal ICU, she had stable course until four months of age, when she was readmitted due to another seizure activity, involving her upper extremity. Her blood glucose level at this time was 84 mg/dL, so this seizure was not thought to be associated with hypoglycemia as opposed to the last one. The EEG was repeated, which revealed foci of epileptiform discharges in the posterior quadrants along with global cerebral dysfunction. Therefore, she was started on daily antiepileptic medications. During this hospitalization, ultrasound of kidney was repeated, which showed interval resolution of previously demonstrated left hydronephrosis, hydroureter, and left ureterocele, so antibiotic prophylaxis was stopped. She will be followed up as outpatient.

## 3. Cytogenetic Study

A karyotype study showed female karyotype with possible deletion of short arm of chromosome. Subsequently, chromosomal microarray analysis was performed by CMA-SNP (single nucleotide polymorphism) V2 method which confirmed the loss of chromosomal region 20p12.1p11.21, spanning approximately 8.348 Mb from linear sequence location 15841592bp to 24189610bp x1 nucleotide position based on hg18. There are more than 20 genes in this region. This deletion does not involve the JAG1 region, associated with Alagille syndrome. No increased regions of absence of heterozygosity (AOH) suggestive of uniparental disomy (UPD) or consanguinity were detected.

## 4. Discussion

Chromosome 20p deletions are rarely seen. Chromosome 20p deletions are associated with a wide variety of anatomical and developmental features. Most of the reported deletions involve 20p12 deletions associated with Alagille syndrome, which is characterized by intrahepatic biliary ductal hypoplasia, peripheral pulmonary artery stenosis, a typical facies with deep set eyes, bossed forehead, posterior embryotoxon of the eye, and vertebral defects [1–3]. Most of the patients with a 20p deletion reported in literature had one major feature of Alagille syndrome. In our patient, however, the deletion does not involve the JAG1 critical region associated with Alagille syndrome; this abnormality is not expected to result in the classic Alagille phenotype [4].

Panhypopituitarism with 20p deletion is uncommon. In reviewing the literature, we have not found any other patients with the exact breakpoints described in our report. However, there are individuals with similar overlapping interstitial deletions of 20p region. In the literature, so far, we have encountered three other reported cases with 20p deletion and panhypopituitarism [4–6].  Table 1 compares the clinical phenotype of our patient with these individuals. Most of these patients have cognitive delay in development, whereas our patient has global developmental delay. Flat nasal bridge, retrognathia, and ear abnormalities are commonly observed craniofacial dysmorphisms. Less common craniofacial dysmorphisms include cleft lip, cleft palate, and epicanthal folds. Vertebral and skeletal anomalies can be seen in 20p11 deletion [5, 7].

Heart anomalies are also seen in Williams et al. [5] along with our patient. Both cases have systolic heart murmur and peripheral pulmonic stenosis. Williams et al.'s [5] case has patent foramen ovale, whereas our patient has patent ductus arteriosus with left to right shunt and biventricular enlargement. Gastrointestinal anomalies are seen in our case along with Williams et al. [5], where it is reported that patient had hiatal hernia, whereas our patient has situs inversus and polysplenia. Genitourinary anomalies were noticed in our case and in Garcia-Heras et al. [6], where it is reported that patient had small phallus, whereas our patient has bilateral enlargement of kidneys, left hydronephrosis with a duplicated collecting system, and obstruction of the upper pole moiety by ureterocele, causing moderate hydroureteronephrosis.

Abnormal brain imaging was seen in all 4 cases. MRI in William et al.'s [5] case showed hypoplastic pituitary gland but there was no evidence of midline fusion of cerebral hemispheres or thalami. MRI in Garcia-Heras et al.'s [6] case showed undescended posterior pituitary gland and cavum septum pellucidum. Kamath et al.'s [4] case number 20 has empty sella on CT scan. Our patient's MRI showed congenital absence of the pituitary gland, with neurohypophysis and adenohypophysis.

Seizures were noticed in Williams et al.'s [5] case along with our patient. Although hypoglycemia and electrolyte abnormalities as part of panhypopituitarism can cause seizures, seizures were not observed in Williams et al.'s [5] and Kamath et al.'s [4] cases which have overlapping deletions with our case, so likely underlying severity brain anomalies might cause increased risk of seizures. As our patient's initial EEG was not diagnostically helpful as the repeat EEG which showed epileptiform discharges, it would be important to do detailed seizure work-up and repeat EEG after 6 to 8 weeks from first seizure episode.

20p deletion may be due to uniparental disomy involving chromosome 20 [8]. Kamath et al. [4] studied cases with deletions involving the region extending from 20p11.21 to 20p12.3. These deletions were found to be de novo in 12 out of 21 patients, maternally inherited in 2, and paternally inherited in 2, and the parents were unavailable for study in 5 cases.

The cause of congenital panhypopituitarism is not known. It has been suggested that the proximal region of 20p carries genes important for midline brain development. Prognosis of these cases would be highly variable and directly related to the severity of the congenital malformations, cardiac involvement, and panhypopituitarism [1]. Seizures, developmental delay, GI malformation, GU malformation, and congenital heart disease may cause significant morbidity.

## 5. Conclusion

As reports of interstitial deletions on the short arm of chromosome 20 are rare, interstitial deletion of 20p associated with panhypopituitarism is further uncommon entity. With this report, we hope to contribute to creating a phenotypic profile including certain dysmorphic features, multiorgan involvement, and related clinical characteristics in this patient population to identify patients with similar interstitial deletion sequences of the 20p region and aid in early diagnosis and interventions for associated comorbid conditions.

## Figures and Tables

**Table 1:** Clinical features in patients with panhypopituitarism and interstitial deletions of 20p11 overlapping regions.

Characteristic traits	Present study	Williams et al., 2011 [5]	Garcia-Heras et al., 2005 [6]	Kamath et al., 2009 [4], case # 20
Chromosomal deletion	20p11.21p12.1	20p11.21p11.23	20p11.1p12	20p11.1p12.2
Gender	Female	Female	Male	Male
Panhypopituitarism	+	+	+	+
Cognitive delay	+	+	NS	+
Congenital heart disease	+	+	−	−
Gastrointestinal system anomalies	+	+	NS	−
Genitourinary system anomalies	+	NS	+	−
Hypoglycemia	+	NS	+	NS
Abnormal brain imaging study	+	+	+	+
Seizures	+	+	NS	NS
Flat nasal bridge	+	+	+	−
Cleft lip/palate	−	+	−	NS
Retrognathia	+	−	+	NS
Low set ears	+	+	NS	NS
Preauricular pits	−	+	+	NS
Vertebral defects	−	+	−	−

NS: not specified.
